# Aphorisms, Definitions, Reflections and Paradoxes—Medical, Surgical, and Dietetic

**Published:** 1902-09

**Authors:** 


**Aphorisms, Definitions, Reflections and Paradoxes — Medical, Surgical, and Dietetic.** By A. Rabagliati, M.D. Pp. xiv., 291. London: Baillière, Tindall and Cox. 1901.

"I dedicate these thoughts," says the author, "resulting in an attempt to simplify the Theory and Art of medicine, to my colleagues of the Medical Profession." This "consummation devoutly to be wished" is, however, not attained to our satisfaction. We begin, in the preface, to be discouraged by reading that we seem no nearer conclusions as to the causation of diseases than we were 2,300 or 2,400 years ago, and soon come to think that this must be so; for we are told that "blood poisoning and septic action in wounds depend more on the state of the patient than on what is done to him at the time of operation"; that "the presence of dropsy must be taken as evidence of excess of lymph in the body, therefore of excess of chyle, and therefore of excess of food"; that "inflammations are the states of the body which result when it reacts against inorganic irritants like cold, heat, wetness, dryness, fatigue, anxiety, and the like," &c. The question of the hereditary transmission of disease is settled offhand: "A child of eight years of age, the son of a drunkard, was found after death to have a hobnail liver." All this, of course, may "simplify" the art of medicine; but this is carried still further when we are told, in aphorism 266, "To the man who knows better, therefore, it is disgraceful to have influenza and pneumonia, or influenza and marked brachycardia!" How many of us must be in disgrace!

Perhaps, however, the summit of "simplification" is reached on p. 147, where the author lucidly remarks: "The states both of *strictum* and *laxum*, both of τάσις and χάλασις, both of shrinking and swelling, both of izanism and œdanism, are induced by *contraction* either of one set of elements or of the other."

Even such ailments as "corns" are not forgotten: "If therefore you suffer from corns . . . these things are marks of poly-siteism and pollaki-sileism" (p. 185). It may be explained that pollaki-sileism is not too much dancing, but merely means eating too often. Indeed, simple words are avoided as much as possible; for example, on p. 186 is a description of what sounds like typhoid fever. It is called *hæmatitis*, and it is pointed out: "In this state anæmic (triphthæmic or catatribæmic?) women become violently feverish [&c.]."

All the paradoxes and aphorisms depend on the assumption that nearly every disease is caused by injudicious diet; and it is perhaps a good thing to have someone who will preach against over-eating and over-drinking, even if the sermon is delivered in the quaint and exaggerative manner of this book.

